# Perforation cæcale néonatale inaugurale du diagnostic d’un mégacôlon congénital

**DOI:** 10.11604/pamj.2018.31.216.15220

**Published:** 2018-12-03

**Authors:** Abdoulaye Diallo Harouna, Idrissa Salahoudine, Abdelhalim Mahamoudi, Aziz El Madi, Khalid Khattala, Youssef Bouabdallah

**Affiliations:** 1 Service de Chirurgie Pédiatrique Viscérale et Urologique CHU Hassan II-Fès, Maroc; 2 Université Sidi Mohamed Ben Abdellah, Faculté de Médecine et de Pharmacie de Fès, Maroc

**Keywords:** Occlusion néonatale, pneumopéritoine, perforation cæcale, mégacôlon congénital, Neonatal occlusion, pneumoperitoneum, caecal perforation, congenital megacolon

## Abstract

Les perforations intestinales spontanées sont rares chez un nouveau-né à terme. Nous rapportons le cas d'un nouveau-né issu d'une grossesse de 41 semaines d'aménorrhée, qui avait présenté à trois jours de vie, un pneumopéritoine inaugural d'un mégacôlon congénital. L'exploration chirurgicale avait mis en évidence une perforation cæcale diastatique associée à une disparité de calibre recto-sigmoïdienne. La prise en charge avait consisté à la réalisation d'une cæcostomie d'urgence après lavage de la cavité péritonéale. L'examen histologique des fragments biopsiques avait permis de confirmer le diagnostic du mégacôlon congénital. Les suites opératoires étaient simples et la cure radicale avait eu lieu six mois plus tard.

## Abstract (en)

Spontaneous intestinal perforations are rare in a full term new-born. We report the case of an infant born at 41 weeks’ gestation who, three days after birth, had pneumoperitoneum revealing congenital megacolon. Surgical exploration showed diastatic perforation of the cecum associated with a disparity in rectosigmoid caliber. The patient underwent emergency caecostomy after peritoneal washing. Histological examination of the biopsy specimens confirmed the diagnosis of congenital megacolon. The postoperative course was simple and radical treatment was performed six months later.

## Introduction

Les perforations intestinales néonatales spontanées surviennent essentiellement chez les grands prématurés. Elles peuvent être à type de perforations intestinales focales idiopathiques ou bien secondaires à une entérocolite ulcèro-nécrosante [1, 2]. Les perforations digestives conduisant au diagnostic d'un mégacôlon congénital (maladie de Hirschsprung) sont exceptionnelles, avec une incidence estimée entre 3,2 à 4,4% selon les auteurs [3]. La perforation cæcale est une complication inhabituelle du mégacôlon congénital [4]. Nous rapportons le cas d'une perforation cæcale diastatique chez un nouveau-né à terme associée à un mégacôlon congénital.

## Patient et observation

**Figure 1:**
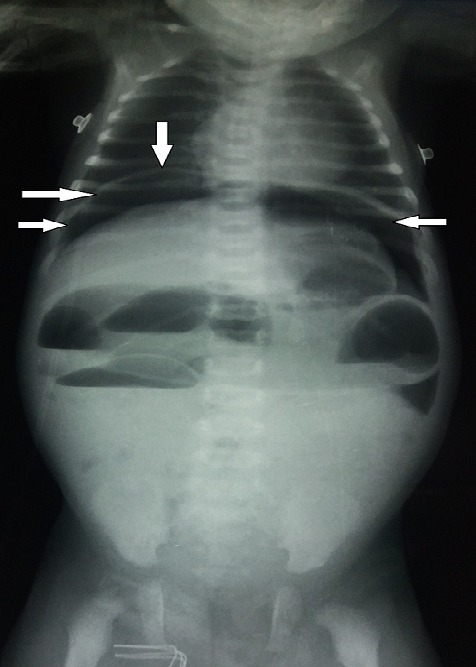
radiographie thoraco-abdominale montrant plusieurs niveaux hydro-aériques sans aération rectale avec un pneumopéritoine massif

**Figure 2:**
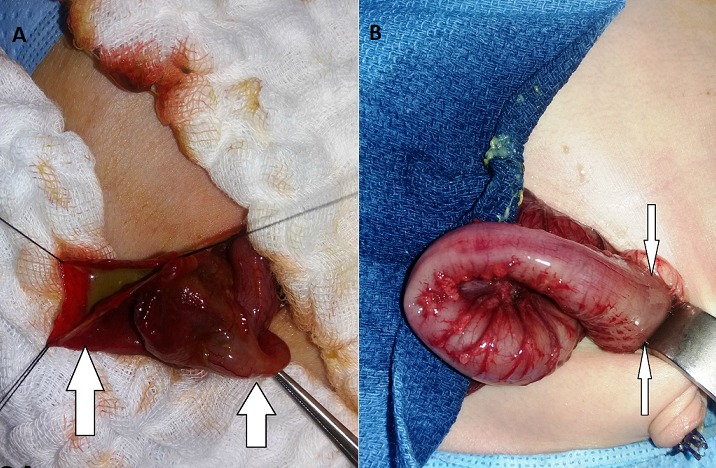
perforation caecale diastatique sur le bord anté-mésentérique (A), présence d’une disparité de calibre rectosigmoïdiènne (B)

**Figure 3:**
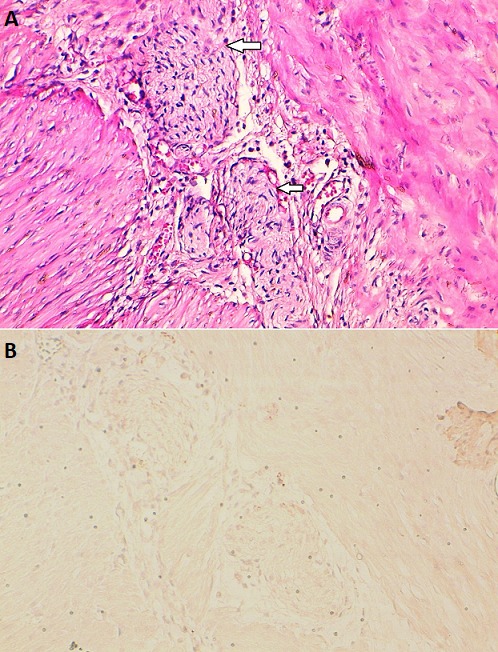
HESx400:mégacolon congénital. Hyperplasie schwannienne manifeste entre les couches de la musculeuse, avec absence de cellules ganglionnaire à ce niveau (A), l’immuno-marquage par l’anticorps anti-calrétine était négatif (B)

Il s'agit d'un nouveau-né de sexe masculin âgé de trois jours de vie, qui a été hospitalisé à l'unité de réanimation néonatale pour une occlusion néonatale. Issu d'une grossesse normale de 41 semaines d'aménorrhée avec un accouchement par voie basse en milieu hospitalier. Le poids à la naissance était de 3400g, avec un score d'Apgar à 10/10 respectivement à 1 minute, 5 minutes et 10 minutes. La symptomatologie a débuté 24 heures après la naissance, par la survenue de refus de téter et de vomissements bilieux. L'évolution était marquée par l'installation progressive d'une distension abdominale et une absence d'émission de méconium. Il était fébrile à 38,2°C, polypnéique à 66cycles/minute, avec une fréquence cardiaque à 130battements/minute, et une SaO2 à 100% à l'air ambiant. L'examen physique notait une distension abdominale tympanique avec une déshydratation modérée. L'épreuve à la sonde rectale était positive ramenant des selles d'aspect normal. Devant le tableau de syndrome occlusif, avec une épreuve à la sonde positive, le diagnostic de mégacôlon congénital avait été évoqué. La radiographie thoraco-abdominale avait objectivé plusieurs niveaux hydro-aériques sans aération rectale. L'association à un pneumopéritoine massif nous a orienté vers le diagnostic d'une perforation diastatique compliquant la maladie sous-jacente (Figure 1). Le bilan biologique préopératoire avait mis en évidence une leucopénie à 6650 éléments blancs/ul et une protéine C réactive à 6 fois la normale. Il s'y associait à une insuffisance rénale d'allure fonctionnelle avec une réserve alcaline effondrée. Après une réhydratation et la correction des troubles ioniques, une laparotomie exploratrice avait permis de découvrir une péritonite stercorale sur perforation diastatique du cæcum (Figure 2A). Le reste de l'exploration du tube digestif avait retrouvé une disparité de calibre rectosigmoïdienne au niveau de laquelle une biopsie étagée avait été réalisée (Figure 2B). Le geste avait consisté à un lavage abondant de la cavité péritonéale, suivi d'une caecostomie. Les suites opératoires étaient simples avec une reprise du transit à J+1 et début d'alimentation orale à J+3. L'examen histologique des fragments biopsiques avait confirmé le diagnostic du mégacôlon congénital (Figure 3). La cure radicale avait eu lieu six mois plus tard et le rétablissement de la continuité digestive 3 mois après. Le suivi post opératoire à moyen terme était sans particularité avec une reprise d'une dynamique digestive normale.

## Discussion

L'incidence des perforations digestives inaugurant une maladie de Hirschsprung oscille entre 3,2 et 4,4% selon les auteurs [3]. Sur le plan physiopathologique, ces perforations surviennent sur un segment intestinal distendu par les gaz et la stase stercorale, responsable d'une pullulation microbienne et d'ischémie pariétale fragilisant ainsi la paroi digestive jusqu'à la rupture de cette dernière [4, 5]. La présentation clinique classique de la maladie de Hirschsprung à la naissance est le retard d'évacuation du méconium au-delà de 24heures suivie d'une occlusion néonatale basse. Devant tout retard d'émission du premier méconium chez un nouveau-né à terme sans autre pathologie connue doit faire évoquer le diagnostic afin d'éviter l'évolution vers des complications comme l'entérocolite et surtout la perforation digestive comme le cas de notre observation. L'épreuve à la sonde est une étape essentielle dans la démarche diagnostique mais aussi thérapeutique en permettant d'apprécier l'aspect normal des selles. Elle peut être utilisée à domicile par les parents qui sont éduqués dans le sens de soulager le bébé après chaque tété [6, 7]. Cependant, devant la persistance d'un ballonnement abdominale tympanique, malgré une épreuve à la sonde répétée et efficace, le diagnostic d'une perforation digestive doit être évoqué conduisant ainsi à la réalisation d'une radiographie thoraco-abdominale de face. En effet, l'existence d'un ballonnement abdominal, luisant et douloureux, survenant quelques heures seulement après la naissance, surtout associé à des vomissements de plus en plus fréquents, verdâtres ; avec des selles liquides et nauséabondes doit faire évoquer en premier le diagnostic d'une entérocolite [6-8].

Dans le cas précis de notre patient, l'analyse de la radiographie thoraco-abdominale nous a permis de mettre en évidence la présence des niveaux hydro-aériques plus larges que hauts en rapport avec une occlusion. L'association à un pneumopéritoine est un signe péjoratif qui témoigne généralement d'une perforation colique. Il ne faut pas oublier l'absence d'aération rectale qui est un signe radiologique classique dans le mégacôlon congénital. Une fois le diagnostic évoqué la prise en charge commence d'abord au lit du patient au moyen d'une exsufflation du pneumopéritoine massif. La technique consiste à introduire un cathéter veineux au niveau de la région sous xiphoïdienne chez un malade en décubitus dorsal permettant de diminuer l'hyper pression abdominale, améliorant ainsi la dynamique respiratoire. Il s'agit d'un geste simple mais qui peut sauver le malade. Le bilan sanguin, montre généralement un syndrome inflammatoire biologique, associé souvent à une leucopénie. En absence de diagnostic précoce, l´évolution se fait vers un tableau septique avec défaillance multi-viscérale. Sur le plan anatomo-pathologique, la perforation digestive survient habituellement au niveau du colon droit et particulièrement le cæcum. Il s'agit du segment colique où les forces de tension sont maximales, et la perfusion pariétale est précaire à ce niveau en cas de distension colique [5]. L'étude de Newman et al. [9] montre que les perforations digestives liées à la maladie de Hirschsprung surviennent préférentiellement dans les formes longues ou coliques totales (62%), siègent sur l'appendice ou le côlon droit (85 %), toujours en amont de la zone aganglionnaire dans les formes courtes ou classiques mais généralement en zone aganglionnaire dans les formes coliques totales (84%). Nous avons rapporté une forme de perforation caecale sur une maladie de Hirschsprung localisée au niveau rectosigmoïdienne, à distance de la zone achalasique. Une fois le bilan lésionnel peropératoire établi, la suite de la prise en charge sera dictée par la nature de la perforation et le degré de septicité locale. Cette prise en charge peut varier, d'un simple avivement suivi d'une suture directe de la perforation à une dérivation digestive transitoire, en passant par la résection et anastomose d'emblée [4]. Il ne faut pas oublier la place de biopsie peropératoire étagée, qui est le seul élément permettant d'avoir la confirmation histologique de la maladie sous-jacente. Dans le cas précis de notre patient, les résultats de l'examen histologique confirment le diagnostic du mégacôlon congénital dans sa forme rectosigmoïdiènne. Avec les progrès réalisés ces dernières décennies, dans le domaine de la réanimation néonatale, le pronostic des perforations digestives est de plus en plus meilleur chez les nouveau-nés à terme. Cependant la mortalité est encore élevée en cas de retard de prise en charge et prématurité [10-12].

## Conclusion

Les perforations coliques focales spontanées chez des nouveau-nés à terme sont rares. Elles peuvent inaugurer souvent le tableau de la maladie de Hirschsprung. Ainsi devant toute occlusion néonatale basse, la présence d'un pneumopéritoine permet d'évoquer le diagnostic de perforation cæcale diastatique. La prise en charge ne doit souffrir d'aucun retard, permettant d'éviter l'évolution vers des complications dramatiques.

## Conflits d’intérêts

Les auteurs ne déclarent aucun conflit d'intérêts.
